# A Generalized Linear Model of a Navigation Network

**DOI:** 10.3389/fncir.2020.00056

**Published:** 2020-09-09

**Authors:** Ehud Vinepinsky, Shay Perchik, Ronen Segev

**Affiliations:** ^1^Department of Life Sciences, Ben Gurion University of the Negev, Beersheba, Israel; ^2^Zlotowski Center for Neuroscience, Ben Gurion University of the Negev, Beersheba, Israel; ^3^Department of Cognitive and Brain Sciences, Ben Gurion University of the Negev, Beersheba, Israel; ^4^Department of Biomedical Engineering, Ben Gurion University of the Negev, Beersheba, Israel

**Keywords:** navigation, grid cell, entorinal cortex, generalized linear model, head direction cells, theta oscillation, speed cells

## Abstract

Navigation by mammals is believed to rely on a network of neurons in the hippocampal formation, which includes the hippocampus, the medial entorhinal cortex (MEC), and additional nearby regions. Neurons in these regions represent spatial information by tuning to the position, orientation, and speed of the animal in the form of head direction cells, speed cells, grid cells, border cells, and unclassified spatially modulated cells. While the properties of single cells are well studied, little is known about the functional structure of the network in the MEC. Here, we use a generalized linear model to study the network of spatially modulated cells in the MEC. We found connectivity patterns between all spatially encoding cells and not only grid cells. In addition, the neurons’ past activity contributed to the overall activity patterns. Finally, position-modulated cells and head direction cells differed in the dependence of the activity on the history. Our results indicate that MEC neurons form a local interacting network to support spatial information representations and suggest an explanation for their complex temporal properties.

## Introduction

The way mammals navigate is considered to rely on networks of neurons in the hippocampal formation, which includes the hippocampus, the dentate gyrus, the subiculum, and the medial entorhinal cortex (MEC). The MEC is an extensively investigated area in the brain, and it is considered to encode the position and orientation of the animal (Moser et al., 2017).

Most studies on navigational information representations in the MEC and related areas have characterized the properties of single cells in the form of a tuning curve to space. In other words, correlating the cellular response with spatial variables (e.g., position, head direction, or speed) can indicate how the cells’ firing rates are related to these variables. This approach has led to the discovery of many types of neurons that encode navigational variables and are located in the MEC, including grid cells (Hafting et al., 2005), border cells (Solstad et al., 2008), head direction cells (Taube et al., 1990), speed cells (Kropff et al., 2015), spatial-modulated cells (Fyhn, 2004), and cells with a conjunctive representation of these spatial variables (Sargolini, 2006; Hardcastle et al., 2017). In addition to pure cell types, a study using an unbiased statistical approach recently reported a high degree of mixed selectivity to navigational variables and heterogeneity in the responses of MEC neurons (Hardcastle et al., 2017).

While most experimental efforts devoted to the neural circuit underlying navigation have explored single cell properties, efforts to understand the mechanism of these cellular properties tend to remain on the network level of description. This has resulted in the development of several theoretical models (Zhang, 1996; Fuhs, 2006; McNaughton et al., 2006; Kropff and Treves, 2008; Burak and Fiete, 2009; Couey et al., 2013; Stepanyuk, 2015; Dordek et al., 2016; D’Albis and Kempter, 2017; Monsalve-Mercado and Leibold, 2017; Weber and Sprekeler, 2018) designed to account for the cellular properties of grid and head direction cells in terms of the architecture of neural networks.

One of the key theoretical approaches is the family of continuous attractor models (Zhang, 1996; Fuhs, 2006; McNaughton et al., 2006; Burak and Fiete, 2009; Couey et al., 2013), which predicts the connectivity structure of cells within networks where the firing pattern of head direction and grid cells is modeled using a Mexican-hat connectivity pattern. Here, cells with similar functional properties (e.g., a similar directional preference for head direction cells) excite each other, whereas cells with different functional properties (e.g., opposite directional preference for head direction cells) inhibit each other (Zhang, 1996). This pattern of connectivity in grid cells implies that neurons with a closer phase distance excite each other, whereas those with a larger phase distance inhibit each other (Fuhs, 2006; McNaughton et al., 2006; Burak and Fiete, 2009). A statistical analysis of electrophysiological recordings of grid neurons showed that correlation patterns between cells are consistent with the theoretical prediction (Dunn et al., 2015). However, these findings do not shed light on the detailed structure of the network connectivity patterns. Clearly, to understand how networks of neurons in the MEC encode information, both the spatial variables as well as the interaction between neurons in the network must be taken into account.

**FIGURE 1:**
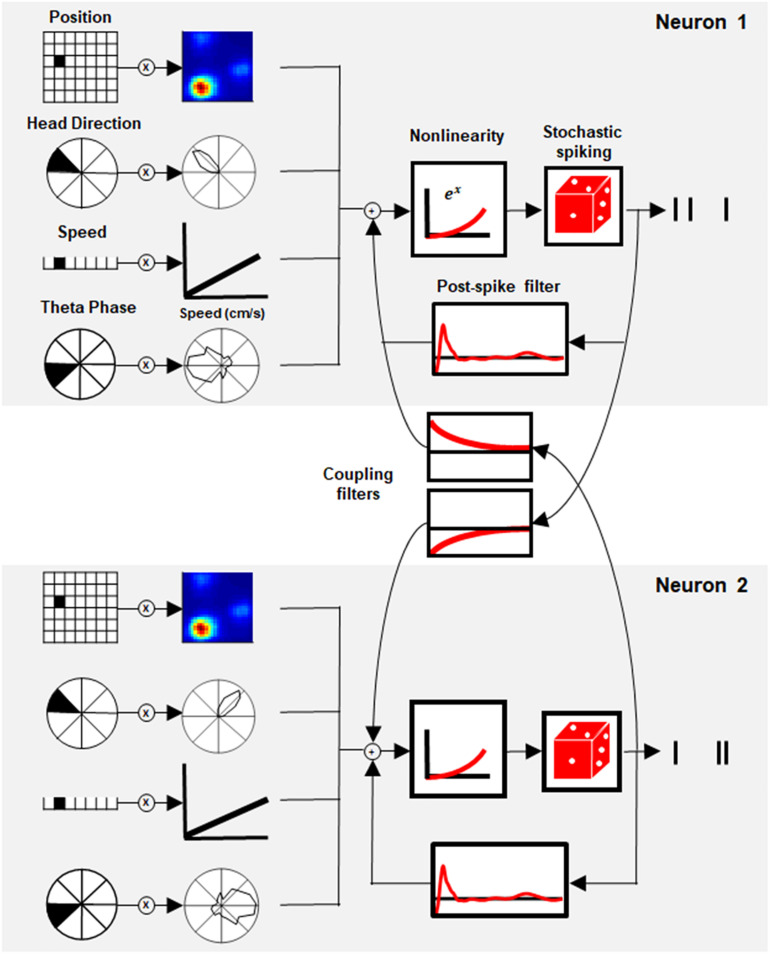
Generalized linear model (GLM) of space representation. Schematic of the GLM framework for two paired neurons. Each neuron’s activity was modeled by a set of stimulus filters (position, head direction, speed, and theta phase) together with the postspike filter and interaction filter between paired neurons. The sum of the stimulus filters’ output with its history and coupling interaction was subjected to exponential non-linearity to generate the instantaneous firing rate of the neuron.

To address this question, we used the generalized linear model (GLM), a flexible generalization of linear regression that makes it possible to capture a response that is non-linear using a link function (Nelder and Wedderburn, 1972). In neural coding, GLM can model the neural response with a set of spatial and temporal filters. Here, we present the *GLM of Space Representation*, a model that includes stimulus-dependent filters of position, head direction, speed, and theta phase, together with temporal filters, which include postspike influence and, most importantly, interactions between neurons (Figure 1).

Although this model is phenomenological by nature, its components can be compared to biophysical mechanisms and used to reveal network structure. The statistical model architecture (see section “Materials and Methods”, Generalized Linear Model), extend the linear–non-linear model (Hardcastle et al., 2017) by taking the temporal correlation within and between neurons into account, as shown earlier in other systems (Pillow et al., 2008; Archer et al., 2014; Park et al., 2014).

Using this model, we study network structure and show that network properties determine the temporal profile of correlations between cells and includes all spatially tuned cells. This result suggests that the interaction between cells extends beyond pure cell types, thus implying that a revision of the prominent models in the field may be in order.

## Materials and Methods

### Data

The data were taken from a publicly available Dataset^1^ (see Supplementary Table 1 for more details) (Sargolini Centre for the Biology of Memory, 2014). The data were collected from rats (*n* = 8) foraging in a 2D environment (100 × 100 cm), while neural activity from the medial entorhinal cortex was recorded using tetrodes. We used 10 min sessions that included the animal’s location, the activity of simultaneously recorded neurons, and the local field potential (LFP). All variables were up-sampled into 1 ms bins. The phase of the theta oscillation was computed from the LFP using the Hilbert transform.

### Code Availability

All codes were written in Matlab and are available at https://github.com/SegevLab/NavigationalGLM.git.

### Generalized Linear Model

The neuron firing rate is modeled by a linear–non-linear Poisson (LNP) model. In this model, the stimulus (*x*) is multiplied by a set of linear filters (*w*) and then is transferred to a static non-linear function (exponent) that gives rise to the instantaneous firing rate. The model can be described as:

(1) $$\begin{array}{c} \lambda_{k}\left(t\right)=\exp[\mu_{k}+\sum_{m=1}^{4}\sum_{i=1}^{L_{\left(m\right)}}w_{i,k}^{\left(m\right)}\cdot x_{i,t}^{\left(m\right)}+\sum_{\tau =t-n}^{t-1}h_{k,\tau }\cdot y_{k,\tau }\\ +\sum_{j\neq k}\sum_{\tau =t-n}^{t-1}l_{j,k,\tau }\cdot y_{j,\tau }] \end{array}$$

where λ_*k*_(*t*) represents the firing rate of neuron *k* at a given time *t*. The term exp (μ_*k*_) describes the baseline firing rate. The expression $\sum_{m=1}^{4}\sum_{i=1}^{L\left(m\right)}w_{i,k}^{\left(m\right)}\cdot x_{i,t}^{\left(m\right)}$ represents the influence of the stimulus on the firing rate of neuron *k*. The term $w_{i,k}^{\left(m\right)}$ denotes a stimulus filter of the neuron, where (*m*) represent the stimulus type (position, head direction, speed, and theta phase), *L*(*m*) is the filter length (625, 30, 10, and 10, respectively). The stimulus at time *t* is represented by a one-hot vector $x_{i,t}^{\left(m\right)}$ in which the index that represents the position at time *t* is set to 1 and all other indices are set to 0.

The expression $\sum_{\tau =t-n}^{t-1}h_{k,\tau }\cdot y_{k,\tau }$ represents the history filter influence on the current firing rate, i.e., the influence of the neuron past activity on the current activity. The term *h*_*k,τ*_ represents the postspike filter of the neuron *k* at past time τ, *y*_*k,τ*_ represents the spike train of neuron *k* at past time τ, and *n* represents the number of past time steps that influence the current neuron activity.

The expression $\sum_{j\neq k}\sum_{\tau =t-n}^{t-1}l_{j,k,\tau }\cdot y_{j,\tau }$ represents the coupling filter influence on the current firing rate. The coupling filter models the influence of the other neurons’ past activity on the current activity of neuron *k*. The term *l*_*j,k,τ*_ represents the interaction strength between neuron *k* to neuron *j* at past time τ, *y*_*j*,τ_ represents the spike train of neuron *j* at past time τ, and *n* represents the number of past time steps that influence the current neuron activity.

The animal’s position in the arena is described by *x*^(*Position*)^, which contains 625 bins, where each bin refers to 16 cm^2^ of the environment. The animal’s head direction is described by *x*^(*Head Direction*)^, which contains 30 bins, where each bin represents 12°. Speed is described by *x*^(*Speed*)^, which contains 10 bins, each of which represents 5 cm/s. The theta phase is described by *x*^(*Theta Phase*)^, which contains 10 bins.

The postspike filter *h*_*k,τ*_ is constructed by a linear sum of a basis of raised cosine “bumps” as described in Pillow et al. (2008). We used 16 basis functions to model the temporal structure of the postspike filter for 150–200 ms. To model the interaction between neurons, we used two types of interaction filters: 1. simple Interaction – which uses one base function, *ce*^−α⋅*t*^, and is active from the third millisecond after the spike and decays slowly until ablation to zero after 54 ms, where *c* is the interaction strength, and we set α = 0.1 for all cells (Supplementary Figure 10A, red line) 2. complex interaction – a set of four bases of raised cosine “bumps” as described in Pillow et al. (2008) (Supplementary Figure 10A, yellow line).

### Model Fit

We fit the stimulus parameters *w*, as well as the history filter *h* and interaction filters *l* using 60% of the data (6 min). This was done by maximizing the log likelihood of the measured cell firing rate *r*_*t*_, given the model’s firing rate λ_*t*_ (Hardcastle et al., 2017; Pillow et al., 2008):

$${\hat{w}}_{k},{\hat{h}}_{k},{\hat{l}}_{j,k},{\hat{\mu }}_{k}=argmax_{w_{k},h_{k},l_{j,k},\mu_{k}}$$

(2) $$\left\{\sum_{t}\log P\left(r_{t}|\lambda_{k}\right)-\sum_{m=1}^{4}\beta_{\left(m\right)}\sum_{i=1}^{L_{\left(m\right)}-1}\left(w_{i,k}^{\left(m\right)}-w_{i+1,k}^{\left(m\right)}\right)\right\}$$

In the left term, *k* resembles the *k*^*th*^ neuron. The rightmost term represents a penalty, which is based on prior knowledge that stimulus parameters should be smooth. The *i* indicator represents an index in the vector *w*^(*m*)^. Parameter β^(*m*)^ represents the smoothing hyperparameter (i.e., a parameter whose value is set before the learning process begins) for variable *w*^(*m*)^, and was chosen for each variable separately but was the same for all neurons. The β^(*m*)^ parameter was chosen by a prior step of parameter tuning. The position parameters were smoothed in two dimensions. Following Hardcastle et al., the parameters were optimized by using MATLAB’s fminunc function. In the training step, model performance was quantified by computing the log-likelihood of held-out data under the model. A K-fold cross-validation procedure was carried out during the training step (10-fold). The parameters for each fold were kept separately, and the mean of the parameters across all folds was considered as the model’s parameters, similar to Hardcastle et al. (2017).

### Model Selection

For each neuron, multiple models were fit. First, simple models with only stimulus information *x*^(*m*)^ were fit to the data. For each neuron, 15 simple models were fit; one model for every variable *i* (position, head direction, speed, and theta phase), one for each combination of two or three variables, and one for all the variables.

To evaluate model performance, we used the same procedure as described in Hardcastle et al. (2017). First, we divided the validation set (20%, 2 min) into sixfold, and for each fold, we quantified the log-likelihood increase from a fixed mean firing rate. To select the simplest model that best described the neural data, we first found the single model with the highest performance. Then, we selected the two variables model with the highest performance that included the selected variable. Next, we compared these models by running a one-sided rank test with a significance value of 0.05. If the more complex model did not perform significantly better than the simpler model, the simpler model was chosen. This procedure was repeated for three and four variables.

After choosing the best model for each neuron, we again ran the training step for this neuron with the selected simple model but now added a postspike filter to the model. This was done for each neuron separately. To take into account the interaction between neurons, we ran the training step one more time with the selected model, postspike filter, and interaction filters. In this way, for each neuron, we had three models: a stimulus filter only model, a postspike filter model, and a full model.

### Model Performance

To evaluate model performance, we used the remaining unused part of the data as a test set (20% of the data, 2 min). For each model (stimulus filter only, postspike filter, and full model), 100 simulated repeats of the spike train were made. We then calculated the poststimulus time histogram in 20 ms bins, smoothed it using a Gaussian filter with a sigma of 40 ms, and then compared it to the experimental data by calculating the correlation coefficient (Kass et al., 2003).

To test whether the model was able to generalize, we used the correlation coefficient between the predicted PSTH and the experimental data. For each model, 1,000 shuffling procedures were conducted, where the PSTH was shifted. For each shift, the correlation coefficient was calculated. For neuron classification, we considered all neurons for which one of their models had a *p*-value below 0.05. For further analysis, only neurons for which all of their models had a *p*-value below 0.05 were included.

### Model Fitting and Data Testing

The fitting procedure was as follows: 6 min was used to train each model. Then, additional 2 min was used for model selection, that is, selecting which model best describes the cells in terms of stimulus filter only, i.e., position, head direction, speed, and theta. Finally, the remaining 2 min were used as test sets to quantify the success of the model. To quantify the success of the postspike filter model in predicting interspike interval (ISI) and autocorrelation, we used the full last 4 min to test the success of the model. In the case of the full model, we also used the last 4 min to test the success in predicting the cross-correlation. We adopted this strategy due to the short data available. However, this strategy can be adopted since the last 4 min was not used to train the model.

### Spike-Train Simulation

The simulation was done by a point process taken from Pillow et al. (2008), using the learned parameters. For the coupling model, we used two types of simulations. When comparing the models’ firing rates, we used the precise spike time of simultaneously recorded neurons and projected them into the interaction filters. To quantify the interaction between neurons, we simulated the neurons in the session simultaneously without the precise spike time from the experiment.

### Classic Grid and Border Scores

The grid and border scores were calculated by first computing the smoothed position tuning curve (Gaussian smoothing filter, σ = 2 cm). Each bin in the position tuning curve corresponded to 2 cm × 2 cm in the environment, and the mean number of spikes/s for each bin was computed. The grid score was calculated using code taken from Ismakov et al. (2017). The grid score was computed as the symmetric rotational score on the 2D autocorrelogram of the position tuning curve (Sargolini, 2006). Neurons with a grid score above 0.5 that passed the null hypothesis test were classified as grid cells. The border scores were computed as $\frac{CM-DM}{CM+DM}$, where *CM* represents the max fraction of bins along a wall of all firing fields, and *DM* is a normalization of the product between the distance of a bin from the nearest wall with the firing rate. The analysis was only done on neighboring bins with a firing rate exceeding the 0.3 max firing rate, covering a total area of 200 cm^2^ (Solstad et al., 2008). Neurons with border score above 0.5 that passed the null hypothesis test were classified as border cells.

The head direction tuning curve was calculated as the mean firing rate in 12 bins (30°). The head direction score was defined as the mean Rayleigh vector length of the tuning curve (Hardcastle et al., 2017). The threshold for the head direction score was set to 0.3.

Speed scores were computed as the correlation between the firing rate (20 ms bins, Gaussian smoothing with σ = 40 ms) and the running speeds (2–100 cm/s) (Kropff et al., 2015). The speed stability was computed as the mean correlation between speed tuning curves (mean firing rate for 5 cm/s bin) using quarters of the session (Kropff et al., 2015). Speed cells were considered neurons that passed both the speed score (above 0.1) and the speed stability test.

To calculate the spatial coherence score, we first computed the position-tuning curve without smoothing for 2 cm × 2 cm bins. The arch tangent of the correlation coefficient between a pixel in the tuning curve and the average of the neighboring pixels for all pixels in the tuning curve was defined as the coherence score (Brun et al., 2008). The threshold for the spatial coherence test was set to 0.5.

### Classic Test Score Threshold

The threshold for distinguishing between cell classes and non-classified cells for each test (grid, head direction, border, speed, and spatial coherence) was computed by taking the 99th percentile of a null (shuffled) distribution of scores. The null distribution of scores was calculated by 1,000 circular shifts of the firing rate of single neurons by a random time shift (minimum 1 s). For each shift, the test scores values were recorded.

### Cross-Correlation Index

The normalized cross-correlation function between two neurons spike trains *y*_1_ and *y*_2_was computed as follows:

(3) $$C\left(\tau \right)=\left(\sum_{t}y_{1}\left(t\right)y_{2}\left(t+\tau \right)\right)/\sum_{t}y_{2}\left(t\right)$$

We calculated the cross-correlation in 10 ms bins, limited for 300 ms around a spike. To get a cross-correlation index, we computed the correlation coefficient between the MEC neurons cross-correlation to the GLM-simulated neurons cross-correlation.

Moreover, we computed the cross-correlation between every two MEC neurons using the train-set data and calculated its correlation with the MEC test-set cross-correlation. This enabled us to compare the raw data estimator (training set cross-correlation) to the GLM estimator (simulated data cross-correlation), either in the full model or one interaction trimmed model.

### Noise Correlation

The position noise correlation was computed by dividing the arena into 4 cm × 4 cm bins, where for each bin, we calculated the correlation coefficient between the firing rate of two neurons, at a 1 ms resolution (Gaussian smoothing with σ = 2 ms). The mean of the correlation coefficient over all bins was considered as the position noise correlation (Cohen and Kohn, 2011).

### Firing Rate Correlation

The firing rate correlation was defined as the correlation coefficient between a pair of neurons’ firing rates, smoothed with a Gaussian window with a sigma of 2 ms.

### Place Field Similarity

Place field similarity was computed using position tuning curves with 4 cm × 4 cm bins, smoothed with a Gaussian window with a sigma of 2 ms. The correlation coefficient between the position tuning curves of two neurons was defined as the place field similarity (Cohen and Kohn, 2011).

### Relative Spatial Phase

The relative spatial phase between two grid cells from the same module was defined as the shift with the highest correlation value in their spatial cross-correlation, as described in Langston et al. (2010). In order to select neurons from the same module, we only used grid neurons whose radius difference was < 10 cm.

### Autocorrelation Index

The autocorrelations of a neuron were computed in the same way as the cross-correlation. The autocorrelation index was defined as the correlation coefficient between two autocorrelation vectors of the MEC and GLM neurons after setting the bin in τ = 0 to zero.

### Interspike Interval Index

The interspike interval distribution was calculated as the histogram of the time difference between two adjacent spikes, at a 1 ms resolution, divided by the number of spikes. The interspike interval index was computed as the correlation coefficient between two interspike interval distributions of the MEC and the GLM neuron.

### Index Comparison

The index comparison was calculated using the test and validation data (40%, 4 min), given the size of the dataset.

### Two Postspike Filters

For each neuron, we used two postspike filters. The first filter was applied only after spikes in which the interval from the previous spike exceeded 35 ms. These spikes can be seen as the first spike in a time window or the first spike in a burst. The second post spike filter was applied for all spikes. In this way, we could differentiate between the first spike in a burst and the subsequent spikes. The inclusion of a second postspike filter was motivated by observation in the retina that additional filter in the GLM allows capturing additional information in the neuronal response (albeit small, Tkačik et al., 2014). Here, we wanted to test whether such an additional filter provides such improvement in the model performance.

## Results

We used the GLM approach to quantify the coding properties of MEC neurons in a 2D square arena. We used online available data recorded from rats (Sargolini, 2006), exploring a 1 m × 1 m environment, used by permission from http://www.ntnu.edu/kavli/research/grid-cell-data. The dataset contains a total of 265 neurons recorded from eight different animals.

The GLM approach is based on the well-known linear non-linear Poisson (LNP) model where the input of each cell is described by a set of linear filters (Figure 1). Each filter acts on one of the stimulus modalities (i.e., position, head direction, speed, and theta phase) and thus represents the tuning curve of the cell in time and space. In addition, a history filter, which acts on the cell’s own activity (also known as a postspike filter), captures the interaction between the spikes of the cell. This mechanism introduces refractoriness into the neuronal activity and long-term adaptation in the spike train. One of the key features of the GLM used here is the existence of coupling between cells in the networks through a set of coupling filters. These filters capture the dependencies on the recent spiking of other cells. Overall, for each neuron, the sum of all inputs (stimulus-based, history and network-based) is transferred to a static non-linear function that gives rise to the instantaneous firing rate (Figure 1, see section “Materials and Methods”, Model Description for details).

To determine the contribution of each component of the model; i.e., the stimulus filters, postspike filter, and interaction filters, the analysis was conducted in an iterative manner such that the complexity of the model increased gradually. First, we fitted a GLM that only included stimulus filters (i.e., position, head direction, speed, and theta phase) and selected which stimulus filters produced the best prediction of activity. Then, we included a postspike filter in the model to obtain a more complete view of the single cell dynamics. Finally, we extended the analysis to the full model where we allowed interactions between cells and studied network properties.

### Representation of Space: GLM Can Capture Single Neurons’ Response Structure

**FIGURE 2:**
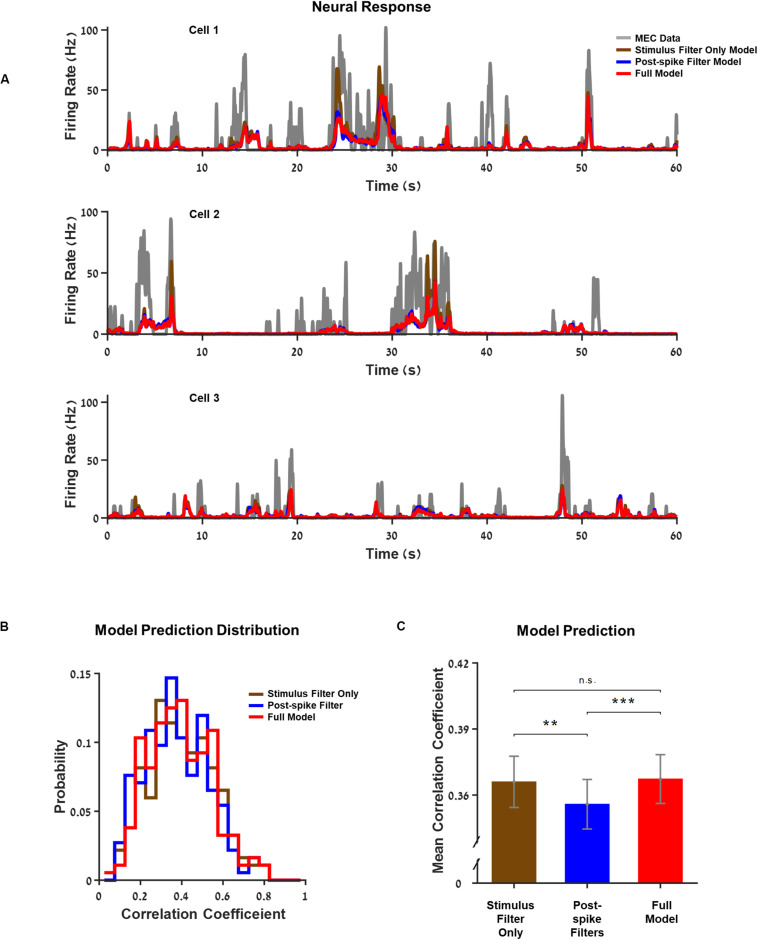
Generalized linear model (GLM) captures single neurons’ response structure. **(A)** Three examples of neurons’ firing rate vs. the models’ predictions over 1 min from the test set. The models increase in complexity from a model based on stimulus filters alone, through a model with a postspike filter to the full model where the interactions between neurons within the network are included. Medial entorhinal cortex (MEC) neurons (gray) compared to GLM prediction: GLM with stimulus filters only (brown), GLM with a postspike filter (blue), and full GLM with interactions (red). **(B)** Histogram of the models’ correlation coefficient: stimulus filter only (brown), postspike filter (blue), and full model (red). The correlation coefficient expresses the extent of the model’s success in predicting the real firing rate of a neuron. **(C)** Average correlation coefficient. The differences between models with different complexities were small. **Indicate *p*_value < 0.05 and *** indicates *p*_value < 0.01.

First, we estimated the quality of the models. Three examples of neuron activity were compared to the prediction of the models with increasing complexity as shown in Figure 2A. The GLM model successfully captured the main firing events but not the detailed structure of individual firing events. However, the differences between the predictions of the firing rate between models that incorporate space variables alone (i.e., position, head direction, and speed) and the model that included a history filter or interactions were minimal. This also emerged clearly when we looked at the population histogram of the correlation coefficients between cellular activity and the predictions (Figures 2B,C and Supplementary Figure 1).

In the following sections, we analyze the differences between the different models in more detail, discuss the ways in which the complex models predict neuronal activity structure better, and what conclusions can be inferred from these results.

### GLM-Based Classification Reveals the Complex Encoding Schemes of Space Variables

**FIGURE 3:**
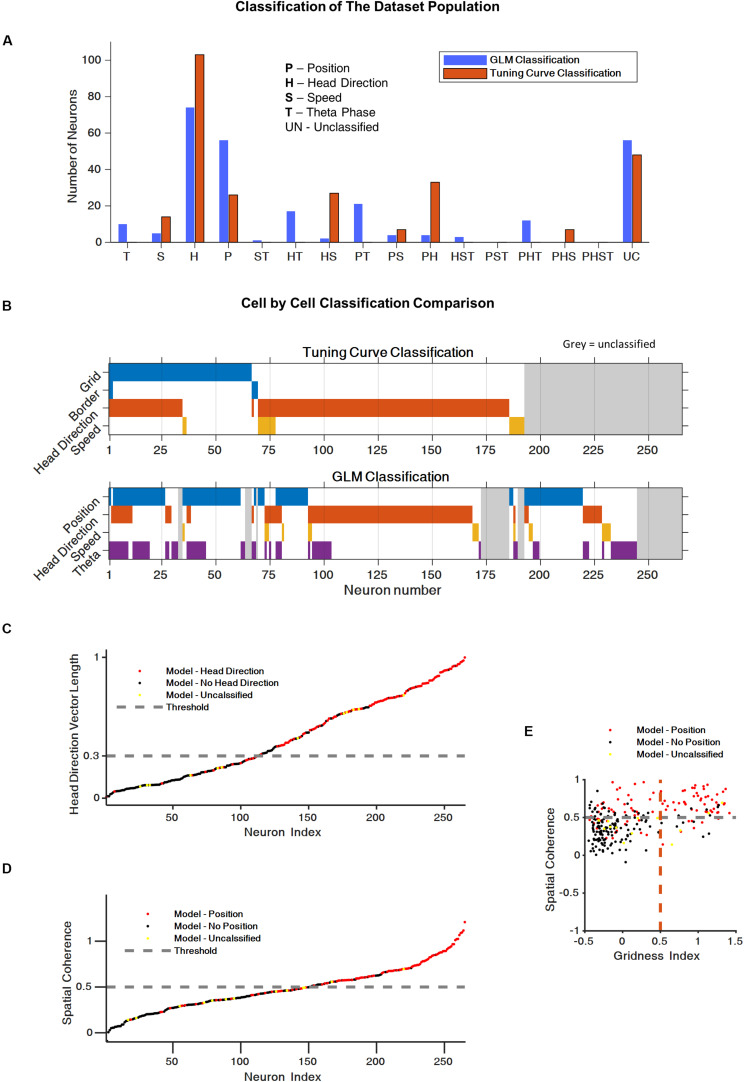
Generalized linear model (GLM) reveals complex encoding scheme of space variables. **(A)** Histogram of the cells population classification using the classical tuning curve approach and the GLM-based approach. **(B)** Cell-by-cell comparison of cell classification using the classical classification and the GLM classification. The color code represent the cell classification; cell marked in gray were unclassified. **(C)** Comparison between the classical head direction classification and the GLM-based classification. Each dot represents a neuron. Head direction classified by GLM (red), non-head direction classified by GLM (black), and non-classified by GLM neurons (yellow). The dashed line represents the head direction score threshold for the classical test. **(D)** Comparison of the classical position encoding (spatial coherence score) classification and the GLM based classification (red = position encoding GLM based, black = non-position encoding, yellow = non-GLM classified neurons, gray dashed line represents the spatial coherence score threshold for classical test). **(E)** Spatial coherence vs. grid score for all cells: neurons that were classified by GLM as position encoding in red, non-position encoding (black) and non-GLM classified neurons (yellow). Gray dashed line represent spatial coherence and grid threshold. The grid cells are a subset of the spatially coherent neurons.

To assess the spatial-encoding properties of the neurons, we used the traditional tuning curve-based classification and the GLM-based approach. First, we used the classical classification for head direction (Hardcastle et al., 2017), speed (Kropff et al., 2015), border (Solstad et al., 2008), and grid encoding (Langston et al., 2010) (see section “Materials and Methods”, Classic Test Scores for details). The classification of neurons using these tests (73% of the neurons) is presented in Figures 3A,B (test scores for whole population are presented in Supplementary Figure 2).

Based on Hardcastle et al. (2017), we then used the GLM-based approach to identify the encoding properties of the neurons that cannot be captured by the classical approach. This approach made it possible to classify 223 of the 265 neurons in the dataset (84% of the neurons). More specifically, the GLM model classify 88% of the traditional classified cells and 80% of the traditional unclassified cells. Figures 3A,B present the coding properties of these neurons. Most of the neurons were classified as position or head direction encoding. In addition, a substantial group of neurons, 24%, encoded two or three space variables, which is indicative of the complex encoding scheme of the population.

A comparison of the classical classification and the GLM-based classification showed that, for most cells, the GLM classification was consistent with the classical method (Figures 3B–E). However, as shown in Supplementary Figures 3–5, the GLM-based response profiles were heterogeneous and captured irregular shapes in the tuning of the neural response to spatial variables.

Finally, we checked whether a model containing three distinct filters for grid, border, and place cells instead of the general spatial filter would outperform the GLM model with one general spatial filter to classify the cells. We found that while this model is good in predicting the firing rate of grid cells, the overall classification is poorer than the one obtained by a general spatial filter (model details and results appear in Supplementary Material and Supplementary Figure 6). To conclude, the model presented here is superior to any other model tested in the overall classification of the entire the cell population.

### A Postspike Filter Increases GLM’s Ability to Predict the Temporal Structure of Single Cell Activity

We extend the best model for each neuron by incorporating the neuron’s history by introducing a postspike filter that is activated whenever the neuron generates an action potential and influences neuronal activity in the near future.

**FIGURE 4:**
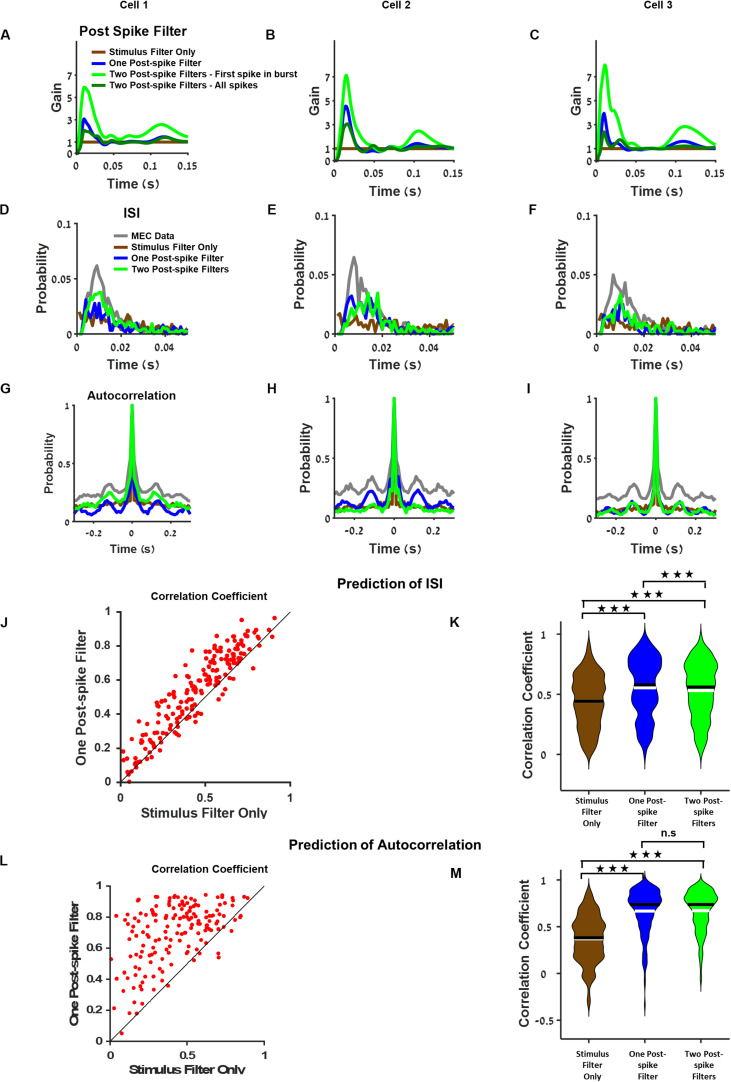
Postspike filter increases prediction accuracy of the temporal structure of single cell activity. **(A–C)** Examples of postspike filters of three cells. The case where the model was based on stimulus filter only is equivalent to a model with a constant postspike filter (brown). The postspike filters capture different aspects of the neuronal dynamics such as bursting and theta oscillations. **(D–F)** Interspike interval of medial entorhinal cortex (MEC) neurons (gray), generalized linear model (GLM) with stimulus filter only (brown), GLM with one postspike filter (blue), and GLM with two postspike filters (green) using filters from **(A–C)**, respectively. **(G–I)** Autocorrelation of MEC neurons (gray), GLM with stimulus filter (brown), GLM with one postspike filter (blue), and GLM with two postspike filters (green) using filters from **(A–C)**, respectively. **(J)** Comparison of the interspike interval index across GLM models. **(K)** Population of interspike interval index for different models. Median (black), mean (white). **(L)** Comparison of the autocorrelation index between GLM models with stimulus filter only and with one postspike filter reveals that one postspike filter increases the accuracy of the prediction of autocorrelation function. **(M)** Population of the autocorrelation index for stimulus filter only (brown), one postspike filter (blue) and two postspike filters (green), median (black), and mean (white). All comparisons were done on held out data.

We compared three types of models in this analysis: 1. a basic model where no postspike filter was used 2. a model with a postspike filter, and 3. a model with two different postspike filters. One filter was used for the first spike in a burst and the other postspike filter for all other spikes in the burst (for the definition of a burst, see section “Materials and Methods”, Two Postspike Filters). The third model tested whether the contribution of the first spike differed from the subsequent spikes. Examples of postspike filters for three sample neurons are presented in Figures 4A–C and Supplementary Figure 7.

The findings showed that introducing a postspike filter increased the model’s ability to predict the temporal structure of the neuronal response. This was apparent by visual inspection of the interspike interval histogram (Figures 4D–F) and in the autocorrelation function of the neuron’s activity (Figures 4G–I). It is worth noting that various amounts of postspike filters constitute a bump after 100 ms (Figures 4A–C) that contributes to the theta oscillatory activity pattern (Figures 4G–I). In addition, in general GLM, firing rate is lower than the MEC neurons firing rate.

Analysis of the population of cells revealed that the model’s ability to predict the interspike interval histogram and the autocorrelation function increased considerably when we introduced a postspike filter (Figure 4J). Specifically, both models with postspike filters outperformed the model with a stimulus filter (Figure 4K, *P* < 0.001). We assessed the similarity in autocorrelations between the data and the model (see section “Materials and Methods”). The models with postspike filters were significantly better in capturing the autocorrelation structure (Figures 4L,M, *P* < 0.001).

Finally, we tested the impact of introducing a second type of postspike contribution to the cellular activity that combined the two postfilters, i.e., a postspike filter for the first spike in a burst and another for all other spikes in a burst (see section “Materials and Methods” for burst definition). However, this did not increase the predictive power of the model substantially (Figures 4K,M and see Supplementary Figure 8 for additional analysis). Thus, a single postspike filter appears to be sufficient to increase the predictive power of the GLM in terms of single cell temporal correlations.

Moreover, we tested whether the prediction power of firing rate is linked to the power to predict the history (i.e., ISI and autocorrelation function). We found that the ability to the predict the ISI profile is linked to the autocorrelation but weakly connected to the ability to predict the firing rate (Supplementary Figure 9).

### Spatially Modulated Cells and Head Direction Cells Differ in the Dependence on History

**FIGURE 5:**
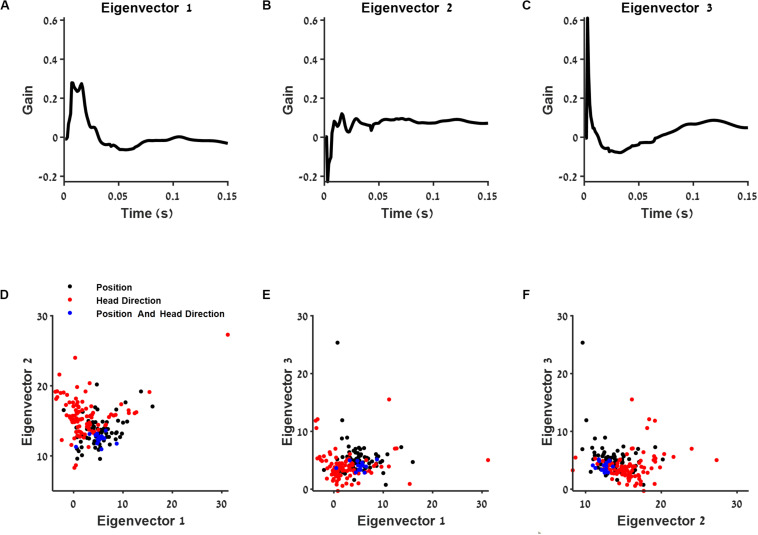
The connection between post-spike filter structure and cell type. **(A–C)** First three eigenvectors of the population postspike filter. **(D–F)** Projection of the postspike filters into two dimensions using the three eigenvectors from **(A–C)**. The projections are shown for position (black), head direction (red), and conjunctive (blue) classified neurons.

To test the dependence of history of different cell types, we calculated the principle components of the entire group of postspike filters of all cells (Figures 5A–C). We found that the first principle component increases both the neuron’s tendency to bursts and oscillations, while the second principle component contributes for bursts alone.

Then, we projected each postspike filter on the three principal components with largest variance (Figures 5D–F). We found that this subspace of the postspike filter of spatially modulated cells differed in the contribution of history from the group of head direction cells (Figure 5D). Specifically, the position-modulated cells tended to burst and oscillate more than the head direction cells.

### Analysis of Interactions Between Neurons

**FIGURE 6:**
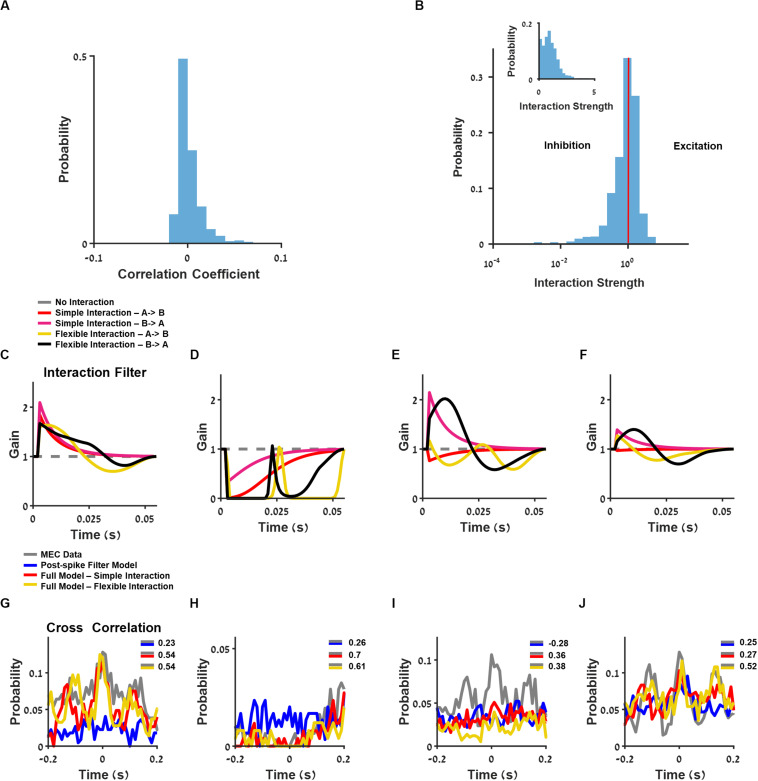
Analysis of interaction between neurons. **(A)** Histogram of neuron pairs’ correlation coefficients. **(B)** Log-scale histogram of the learned interaction strength using the full model with one-base function interaction filter. Linear-scale histogram is presented as inset. **(C–F)** Interaction filters of four pairs of neurons, using full generalized linear model (GLM): one-base function interaction filter (red and pink) and flexible interaction filter (yellow and black). **(G–J)** Cross-correlation between two medial entorhinal cortex (MEC) neurons (gray) and GLM-simulated neurons composed of postspike filter (blue) and full model composed of one base-function interaction filter (red) and flexible interaction filter (yellow), using filters from **(C–F)**.

To reveal the network connection properties, we first measured the firing rate correlation between neurons (Figure 6A) and found that most of the simultaneously recorded neurons were weakly correlated. Then, for each recorded neuron, we fit a model that combined stimulus filters (position, head direction, speed, and theta phase), history information (postspike filter), and the spike activity of all other recorded neurons using a temporal interaction filter (section “Materials and Methods”, Generalized Linear Model). In this way, we fit a filter to describe the strength of effective interactions between the neuron and all other neurons in the network. We tested two types of interaction filters: (a) one-base function of exponential form with a fixed decay constant, which could be either positive or negative; (b) a flexible interaction filter with a set of four-base functions (for more information, see section “Materials and Methods”, Generalized Linear Model, Supplementary Figure 10A). Note that the flexible interaction filter could be both with an excitatory lobe and an inhibitory lobe.

The one-base function interaction filter between neurons could be excitatory with an interaction strength larger than one or inhibitory with an interaction strength less than one (Figure 6B). As depicted in the distribution of this model’s coefficients (Figure 6B), most of the interaction coefficients were centered on one, thus yielding no functional interaction between the majority of the recorded neurons. Finally, the interaction filters between neurons could be symmetric (Figures 6C,D, and corresponding cross-correlation between units, Figures 6G,H), asymmetric (Figure 6E; cross-correlation, Figure 6I), or unidirectional (Figure 6F; cross-correlation, Figure 6J).

### The Interacting Model Increases the Predictive Ability of Cross-Correlations Between Cells

Using the interaction filters, we predicted the cross-correlations between neurons and compared them to the model without interactions. To quantify the ability of the model to capture these interaction properties, we calculated a similarity index between the MEC data and the prediction of cross-correlation across all neuron pairs (section “Materials and Methods”, Cross-Correlation Index).

**FIGURE 7:**
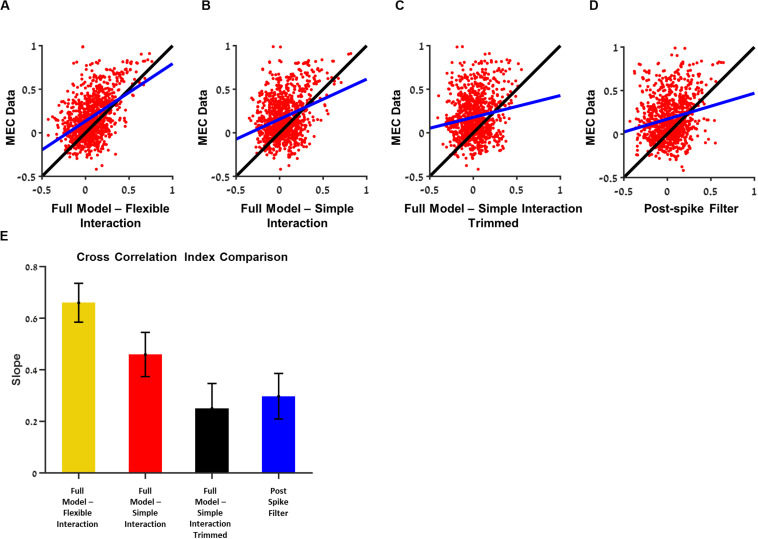
Analysis of the contribution of a single interaction to the ability to predict the cross-correlation between the two cells. Comparisons between the ability of medial entorhinal cortex (MEC) data training set to predict the cross-correlation in the test set with the ability of the generalized linear model (GLM) with the trimmed interaction to predict the. **(A)** Full model with flexible interaction filter. **(B)** Full model with one base function interaction filter. **(C)** Full model with one-base function interaction filter trimmed. **(D)** Postspike filter. **(E)** The slopes of **(A–D)**: when a single interaction is trimmed, the ability to predict the cross-correlation function between the two cells is dropped by factor of about 0.4. All comparisons were done on held out data. The flexible interaction outperformed (*R*^2^ = 0.23, *P* < 0.01) the single-base function interaction (*R*^2^ = 0.1, *P* < 0.01) and both outperformed the non-interacting models (stimulus filter only *R*^2^ = 0.01, postspike filter *R*^2^ = 0.04, *P* < 0.01).

We found that there was a significant improvement in the similarity index when interaction filter between neurons was included in the model (Figures 7A,B). The flexible interaction outperformed the single base function interaction and both outperformed the non-interacting models (stimulus filter only).

Introducing interaction with one-base function improved the cross-correlation index prediction but did not influence the interspike interval index (mean = 0.55) and autocorrelation index (mean = 0.68).

Finally, we tested what is the contribution of the single interaction to the network activity. For this purpose, we trimmed one interaction between two cells at a time and generated a prediction of the activity by the one-interaction trimmed network. Then, we calculated the cross-correlation between the two cells and tested how good was this activity in predicting the actual cross-correlation between cells in the MEC data. Specifically, the quality of this prediction was compared with the ability of the MEC data itself to predict, i.e., the cross-correlation between the two cells in the training set, to predict the cross-correlation between the two cells in the test set.

In summary, the MEC data were then compared with models with full interaction (Figure 7A), full model with simple interaction (Figure 7B), model with one trimmed interaction (Figure 7C), and postspike filter model (Figure 7D).

Using this approach, we were able to test whether the single interaction is critical in predicting the cross-correlation between cell pairs. We found that the eliminating one interaction at a time brought the ability to predict the cross-correlation between the two cells, which were separated to the level of no-interaction (i.e., stimulus only) models (Figure 7E). Therefore, we conclude that the interactions are critical in predicting the cross-correlation between units.

### Dependence of Grid Cells Connectivity on Spatial Phase Difference

**FIGURE 8:**
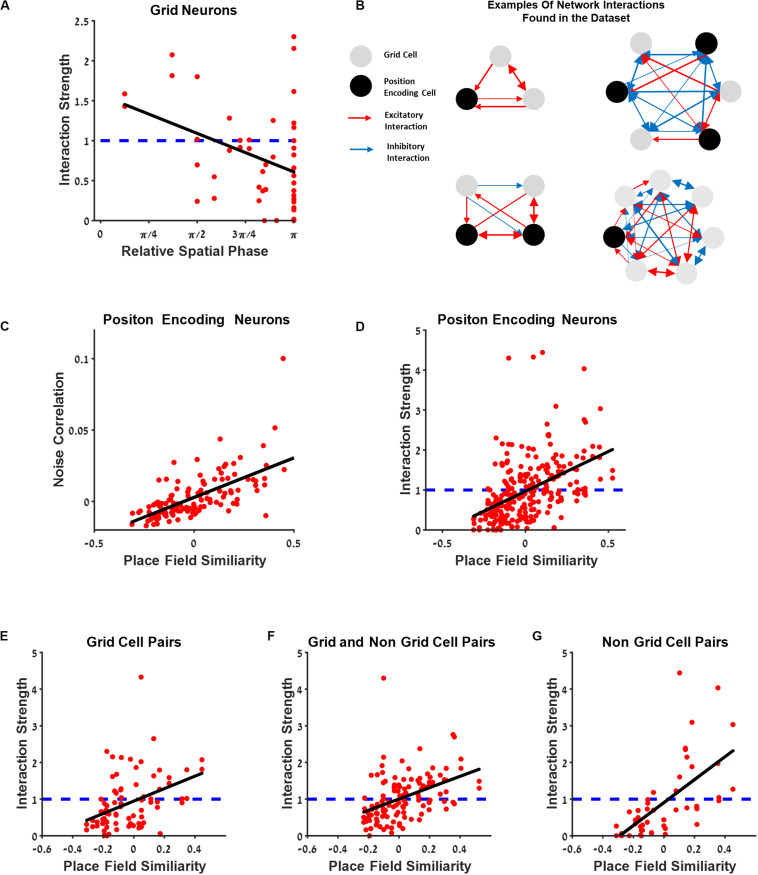
The connectivity between cells extends beyond the grid cell group. **(A)** Generalized linear model (GLM) functional interaction strength vs. grid neurons’ relative spatial phase reveals that grid cells that are functionally near each other have stronger interaction filters. **(B)** Several examples of a schematic of network interactions between the position encoding neurons found in the dataset. Grid neurons (gray) and spatially modulated neuron (black) connected by excitatory and inhibitory interactions (red and blue lines), where thickness represents connection strength. **(C)** Dependence of noise correlation on place field similarity is consistent with previous findings. **(D)** The dependence of the full GLM functional interaction on place field similarity; dashed blue line represents no interaction. **(E–G)** Comparison between place field similarity and full GLM functional interaction strength for grid pairs **(E)**, grid and non-grid pairs **(F)**, and non-grid pairs **(G)**.

Figure 8A presents the relationship between the interaction strength and relative spatial phase between grid neurons. The interaction between cells was high for cells with a small relative spatial phase.

### The Connectivity Between Cells Extends Beyond the Grid Cell Group

To further investigate the connectivity between neurons and to generalize the results obtained within the grid cell population, we examined the interaction strength between all position-encoding neurons (Figure 8B). Naturally, relative spatial phase cannot be used as a measure for a functional distance between cells since the population in this case contains non-grid cells. Thus, we used place field similarity as a measure of the similarity between the space-encoding properties of neurons (see section “Materials and Methods”). The place field similarity is defined as a Pearson correlation between the position tuning curves of the two cells. The analysis was performed on all position-encoding neurons, non-grid cells, and grid cells.

The findings indicated that the interaction strength was high, i.e., excitatory, for cell pairs with high place field similarity, and low, i.e., inhibitory, for cell pairs with low place field similarity (Figure 8D). To further disentangle the contribution of different cell types, we divided the cell pairs into three groups: grid cell pairs (Figure 8E), a grid cell and a non-grid cell pairs (Figure 8F), and non-grid cell pairs (Figure 8G). There was a similar relationship between place field similarity and interaction strength for all groups (Figures 8F,G). This result extends the interaction between neurons as which were found in previous studies not only for grid cells but also for all spatial-modulated cells along with grid cells in the MEC. Finally, we also compared the correlation between place field similarity and noise correlation (see section “Materials and Methods”) and found that a higher similarity of place fields resulted in a higher noise correlation (Figure 8C), suggesting that neurons with closer spatial receptive fields tend to fire together, in line with previous findings (Dunn et al., 2015; Tocker et al., 2015).

## Discussion

The network within the MEC contains grid cells, which encode navigational information in the form of firing fields that are organized in a regular periodic structure, and additional cells that encode space with non-periodic firing fields. Whereas there is a general consensus that the network within the MEC is critical for navigation, the functional connectivity between cells in the MEC remains enigmatic, including the connectivity between grid–grid cells, the non-grid cell population, and across populations. The influence of the recent past activity of neurons on their future activity has also not been characterized. Here, we used a novel theoretical framework to identify how these different contributions determine the network activity of neurons in the MEC.

Using the GLM approach to capture the dynamics of neurons in the MEC, we captured the effective interaction between neurons as well as the temporal correlations of neurons, including the theta oscillatory pattern.

We found that stronger functional interactions were correlated to place field similarity in position encoding neurons, suggesting that the interactions between neurons are stronger for closer spatial receptive fields regardless of the functional classification of these neurons into specific classes such as grid cells.

The finding of effective interactions between different functional types might have biological implications. Looking on the known architecture of the connectivity between the postsubiculum, which contain mainly head direction cells, to the MEC, one could argue that head direction cells in the MEC forms a separated information stream from other functional classes such as grid cells. However, our findings might indicate that all cell types in the MEC are functionally connected either by local interaction in the MEC or derived by one spatially input stream.

The second phase of our analysis examined the contribution of recent history to the activity of single cells. We found that the structure of the temporal filters included the refectory period, burst adaptation, and theta oscillation. Some of these components may be implemented physiologically by interneuron circuitry that controls the gain (Buetfering et al., 2014) and by subthreshold membrane potential oscillations (Alonso and Llinás, 1989).

Although the use of interactions and postspike filters improved the ability to predict correlation structure of neuronal activity both in the form of autocorrelations of single cells or cross-correlations between cells, generally, the temporal filters did not enhance firing rate prediction. This result concords with findings from the retina (Pillow et al., 2008). The GLM approach we used, thus, may constitute a flexible way to model the building blocks that govern neural activity, but as described above, it lacks the ability to predict the neural response in full. The complexity of modeling MEC neurons may involve more dimensions and complex interactions between neurons than presented here. More complex models such as deep learning (LeCun et al., 2015) with recurrent connectivity might result in better predictions. However, this comes with the downside of a lack of interpretation of the model in terms of physiology or a way to justify the architecture through physiological and anatomical equivalents.

Two studies have addressed issues similar to the ones raised in this study, namely, how functional interactions between neurons in the MEC depend on the functional distance between cells. Specifically, Dunn et al. (2015) evaluated pairwise correlations between cells using a maximum entropy kinetic pairwise model and studied their functional connectivity. In their analysis, they took the covariations in firing rates due to overlapping fields into account and found that functional connections decayed with the functional distance between cells. In a second study, Tocker et al. (2015) studied noise correlations of cells in the MEC while accounting for similarity in receptive field structure. Both studies concluded that their findings were consistent with the predictions of the continuous attractor model. The current study thus extends these findings since the GLM approach makes it possible to reveal the temporal structure of the interaction and hence identify the temporal profile of the interaction.

In addition to these studies, Kraus et al. (2015) found that spiking history was the strongest predictor of spike rate variation. Here, we found that there is no significant difference between the model with postspike filters and the models without. The difference between these two results are due to the fundamental difference in the input to the two models. In the Kraus et al. case, the recent history of the neuron itself was used to predict the future activity in the specific trial. One can expect that the actual noise present in the network and influence the neuronal variability in the specific trial. In our case, the only input to the cell is the animal trajectory and does not involve the MEC neuron history but the simulated neuron history only.

One need to keep in mind the limitation of the GLM, which does not provide evidence for the connections between neurons. GLM only reveals the effective connectivity between neurons. This interaction can be the result of a direct synaptic connection between the two cells or a third unobserved entity that influences the activity of the two cells. Such entity could be in the form of a third neuron, which forms synaptic connection on the two cells, or a neuron that mediates information from one cell to the second (i.e., one measured neuron synapses the unobserved neuron, which in turn synapses the second observed neuron). The unobserved entity can be even an entire neuronal circuit that is responsible for the observed interaction.

It should be noted that there is a difference in the number of classified cells by the classical approach and the GLM approach in our study and the finding by Hardcastle et al. (2017), specifically 84 vs. 73% in our case and 77 vs. 42% in the Hardcastle data. This can be explained by the fact that the GLM classifies about 80% of cells regardless of their characterization by the classical approach. In the work of Hardcastle et al., only 42% of the cells were classified initially by traditional classification, which leaves large room for improvement in the GLM approach. In our case, 73% of the cells were classified initially by the classical approach, which leaves little room for improvement. The differences in the datasets can be due to small differences in the recording position or due to preprocessing of the data.

Overall, our study provides a new approach to researching MEC neurons. Using the GLM approach, we successfully quantified how each variable affects the neural response. Applying this framework to a complete population of neurons in the MEC could shed light on network interactions and lead to a better understanding of the ways in which neurons with MEC encode navigational information.

## Data Availability Statement

Publicly available datasets were analyzed in this study. This data can be found here: http://www.ntnu.edu/kavli/research/grid-celldata under the tab “Sargolini et al. 2006” (Sargolini Centre for the Biology of Memory, 2014).

## Author Contributions

EV, SP, and RS designed the research, analyzed the data, and wrote the manuscript. All authors contributed to the article and approved the submitted version.

## Conflict of Interest

The authors declare that the research was conducted in the absence of any commercial or financial relationships that could be construed as a potential conflict of interest.

## Footnotes

1 http://www.ntnu.edu/kavli/research/grid-cell-data
