# Supplementary material for: A Generalized Linear Model of a Navigation Network
Source: Front Neural Circuits. 2020 Sep 9;14:56. doi: 10.3389/fncir.2020.00056 (PMC7509173; doi:10.3389/fncir.2020.00056)

Supplementary Material

Code availability
The codes are available at: https://github.com/SegevLab/NavigationalGLM.git

Stimulus only GLM with three distinct spatial filters

In order to make a direct comparison between the GLM classification and classical classification possible, we fitted three different stimulus only GLM with three distinct spatial filters. These filters represent the classical cell types, place cells, grid cells and border cells. This model can be described as:

$$\lambda_k(t) = \exp\left(\mu_k + \sum_{m=1}^{4}\sum_{i=1}^{L_m} w_{i,k}^{(m)} \cdot x_{i,t}^{(m)}\right)$$

Where $\mu_k, w_{i,k}^{(m)}$ and $x_{i,t}^{(m)}$ are the spontaneous firing rate, the stimulus filters, and the stimulus as in equation (1) in the main text. In these models, we require that the spatial filter will be one of the following:

1. Place cell filter in the form of 2 dimensional Gaussian,

   $f_{place}(\varphi_{1,\dots,6}) = \varphi_1 \exp - \left(\frac{((X-\varphi_2)\cos(\varphi_3)) + ((Y-\varphi_4)\sin(\varphi_3))}{\varphi_5}\right)^2 -$

   $\left(\frac{((X-\varphi_2)\sin(\varphi_3)) + ((Y-\varphi_4)\cos(\varphi_3))}{\varphi_6}\right)^2$

2. Grid cell filter in the form of sum of 3 identical planar waves propagating at 60° to each other,

   $f_{grid}(\varphi_{7,\dots,11}) = \varphi_7 \sum_{l=1}^{3} \cos\left(\frac{2\pi}{\varphi_8}(X-\varphi_9)\sin\left(\frac{l\pi}{3}+\varphi_{10}\right) + \frac{2\pi}{\varphi_8}(Y-\varphi_{11})\cos\left(\frac{l\pi}{3}+\right.\right.$

   $\left.\left.\varphi_{10}\right)\right)$

3. Border cell filter in the form of sum of 4 one-dimensional Gaussians which center on the borders,

$$f_{border}(\varphi_{12,\dots,19}) = \varphi_{12}\exp - \left(\frac{X^2}{\varphi_{13}}\right) + \varphi_{14}\exp - \left(\frac{(X - Arena\_Width)^2}{\varphi_{15}}\right) +$$

$$\varphi_{16}\exp - \left(\frac{Y^2}{\varphi_{17}}\right) + \varphi_{18}\exp - \left(\frac{(Y - Arena\_Width)^2}{\varphi_{19}}\right)$$

The φ_q are parameters to be selected by the maximization of the log likelihood of the measured cell firing rate $r(t)$, given the model's firing rate $\lambda(t)$. All other aspect of the model remained the same.

We found that most classically defined grid cells where indeed classified correctly using this model, there were even few head direction cells, and speed cells, which were found to encode grid pattern as well (Supplementary Figure 6A)&C, orange. In addition, the border cell filter were unable to classify the classically defined border cells (Supplementary Figure 6A&C, yellow). Overall, the GLM with three spatial filters were able to classify 72% of the cell compare to 80% classification in the GLM with one general spatial filter. More specifically, the GLM with three spatial filters were able to classify only 56% of the classically unclassified cells. Therefore, while the GLM model with a specific grid cell can be used to classify Grid cell, the GLM with general spatial filter is more robust and classify higher percentage of cells.

Analysis of two Post-spike filters

In order to know more about the temporal properties of the neurons, we collected the learned post-spike filters of all neurons and used principal component analysis (PCA), to find patterns in the filter structure. By examining the first two eigenvectors (Explained 68 of the variance, Supplementary Figure 8A), We have been able to explain the properties of most neurons in the experiment. The first eigenvector (Supplementary Figure 8B), represents neuron's tendency to burst and oscillate in theta phase. The

second eigenvector (Supplementary Figure 8C), represent the strength of inhibition and long excitation.

We found different temporal properties between the head direction and position encoding cells. In the position encoding neurons, the first spike in burst filter, is more influenced by the burst and theta oscillation then all other spikes filter (Supplementary Figure 8D). In the head direction neurons, the difference between first-spike filter to all spikes filter is the sensitivity to both eigenvectors, which is little higher (Supplementary Figure 8E).

Following this observation, we think that the post-spike filter capture both temporal properties but also some hidden network properties, that can explain the bursting and oscillation of a neuron. We suggest that the connectivity in this two types of networks, position and head direction, is built differently and can be better understood by analyzing large scale population recording.

Figure captions for supplementary tables and figures

Supplementary Table 1| Summary of how many cells were recorded simultaneously, how many interaction terms were added and how many distinct sessions there were in the dataset.

Supplementary Figure 1| Comparing prediction of firing rates between models. Comparison of the models' correlation coefficient. The correlation coefficient expresses the extent of the model's success in predicting the real firing rate of a neuron. **A.** stimulus filter only vs. post-spike filter **B.** Stimulus filter only vs. full model. **C.** Post-spike filter vs. full model.

Supplementary Figure 2| Classic tests scores. Histograms of test scores for all neurons that were analyzed, red line represent the test threshold. **A.** Head direction. **B.** Spatial coherence. **C.** Grid **D.** Border.

Supplementary Figure 3| Position filters. Model-derived response profile of all neurons that were classified as position encoding only or conjunctive coding with: theta phase and/or speed.

Supplementary Figure 4| Head direction filters. Model-derived response profile of all neurons that were classified as head direction encoding only or conjunctive coding with: theta phase and/or speed.

Supplementary Figure 5| Conjunctive position and head direction filters. Model-derived response profile of all neurons that were classified as both head direction and position.

Supplementary Figure 6| Classification by Stimulus only GLM with three distinct spatial filters. Cell-by cell comparison of cell classification using tuning curve classification **(A)**, stimulus only GLM **(B)** and Stimulus only GLM with distinct spatial filters for Grid, Place and Border cell **(C)**.

Supplementary Figure 7| Additional examples of post spike filters. A – F. Learned post-spike filters. Two post spike filters: The first spike in burst filter (bright green) and all-spikes filter (dark green). One post spike filter (blue) The dashed line represents no influence of the post-spike filter. **G – L.** Inter spike interval of MEC neurons (gray), stimulus filter only (brown), GLM with one post-spike filter (blue) and GLM with two post-spike filters (green). Using filters from **A – F** respectively. **M – R.** Autocorrelation of MEC neurons (gray), stimulus filter only (brown) GLM with one post-spike filter (blue) and two post-spike filters (green). Using filters from **A – F** respectively.

Supplementary Figure 8| Analysis of two Post-spike filters. A. The percentage of variance explained, when adding eigenvectors of the post spike filters covariance matrix. **B – C.** First two eigenvectors of the population post-spike filters. **D – E.** Projection of the post-spike filters into 2 dimensions using the eigenvectors from **B – C**. The projections are shown for position and head direction GLM classified neurons respectively, For first spike in burst filter (red), and for all other spikes post-spike filter (blue). **F.** Projection of the post-spike filters into 2 dimensions using the eigenvectors

from **B – C**. The projections are shown for position (black) and head direction (red) GLM classified neurons, using first spike in burst filter.

Supplementary Figure 9| Testing whether model's prediction accuracy of the firing of individual cells is consistent across the three domains of firing rate, ISI and autocorrelation. We tested for a possible connection between the cell classification (position neurons or head direction neurons) and the different metrics and. **A and B**. We found no clear connection between the success of the model prediction and cell type. **C-E.** To test whether the model prediction is consistent across the three domains we highlight the top and bottom quarter of each of the metrics used to evaluate the model performance. We found that the ISI index was correlated with the autocorrelation **(D and E)**. However, this is expected due to the connection of the metrics to temporal correlations of the spike trains.

Supplementary Figure 10| Analysis of interaction between neurons. A. Basis functions used to represent the interaction between neurons for the full model: simple (red) and complex (yellow).

Supplementary Table 1 – Vinepinsky & Perchik et al
Data set information

Session name	Number of neurons	Number of interactions
11025-01060511	3	6
11025-19050503	8	56
11025-20050501	7	42
11084-03020501	6	30
11084-08030506	6	30
11084-09030501	6	30
11084-10030502	9	72
11138-06040507	5	20
11138-07040501	3	6
11138-11040509	5	20
11138-12110501	4	12
11138-13040502	3	6
11138-20040502	7	42
11138-26040501	3	6
11207-05070501	3	6
11207-08060501	3	6
11207-10070501	4	12
11207-11060501	12	132
11207-14060501	9	72
11207-16060501	10	90
11207-20060501	7	42
11207-21060503	13	156
11207-23060501	9	72
11207-27060501	14	182
11207-30060501	10	90
11265-01020602	9	72
11265-02020601	11	110
11265-03020601	12	132
11265-07020602	8	56
11265-09020601	13	156
11265-13020601	5	20
11265-31010601	8	56
11278-30080505	6	30
11278-31080502	11	110
11340-01120501	2	2
11340-22110501	5	20
11340-25110501	3	6
11343-08120502	3	6
**Total**	**265**	**2014**

Supplementary Figure 1 – Vinepinsky & Perchik et al

Comparing prediction of firing rate between models

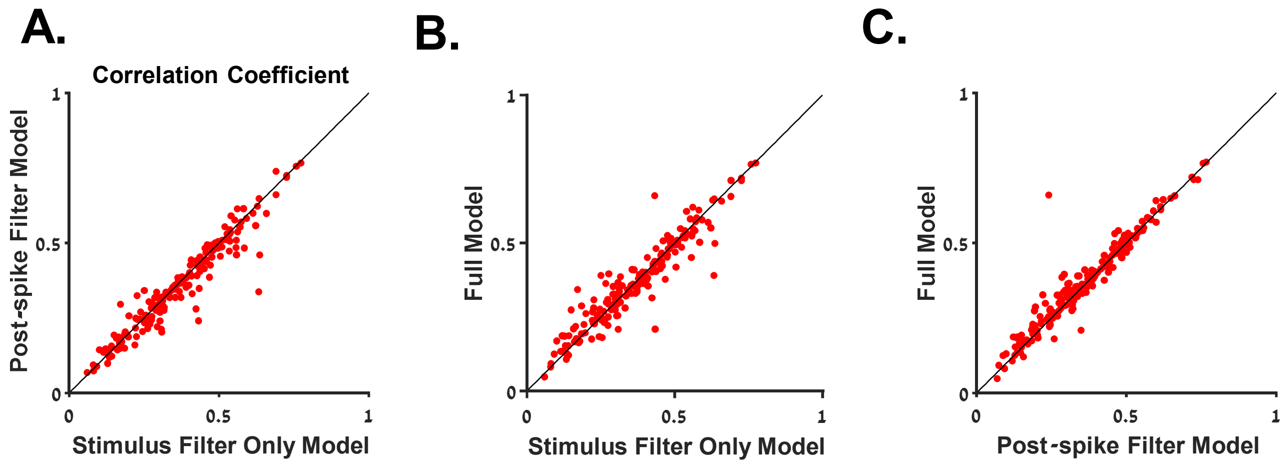

Supplementary Figure 2 – Vinepinsky & Perchik et al

Classic tests scores

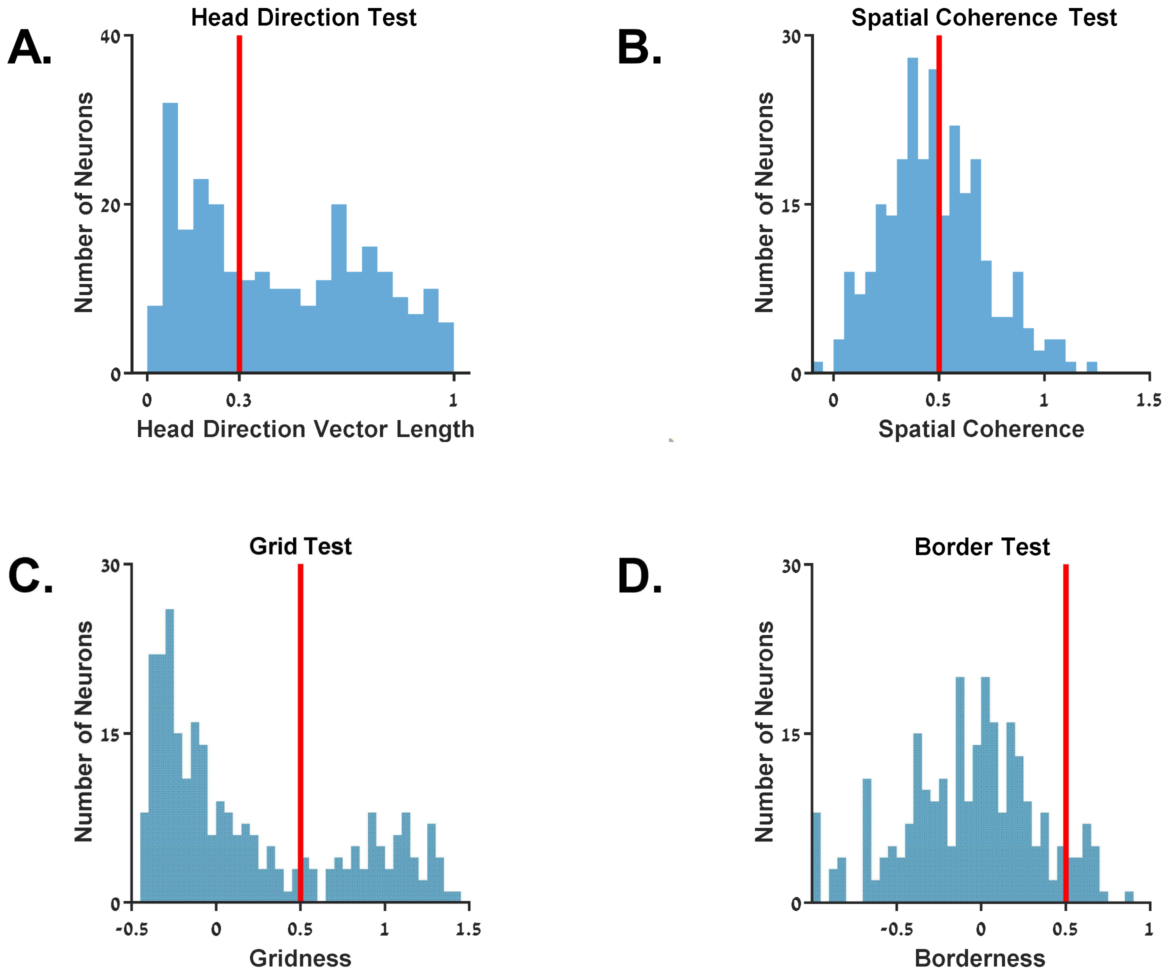

Supplementary Figure 3 – Vinepinsky & Perchik et al

Position filters

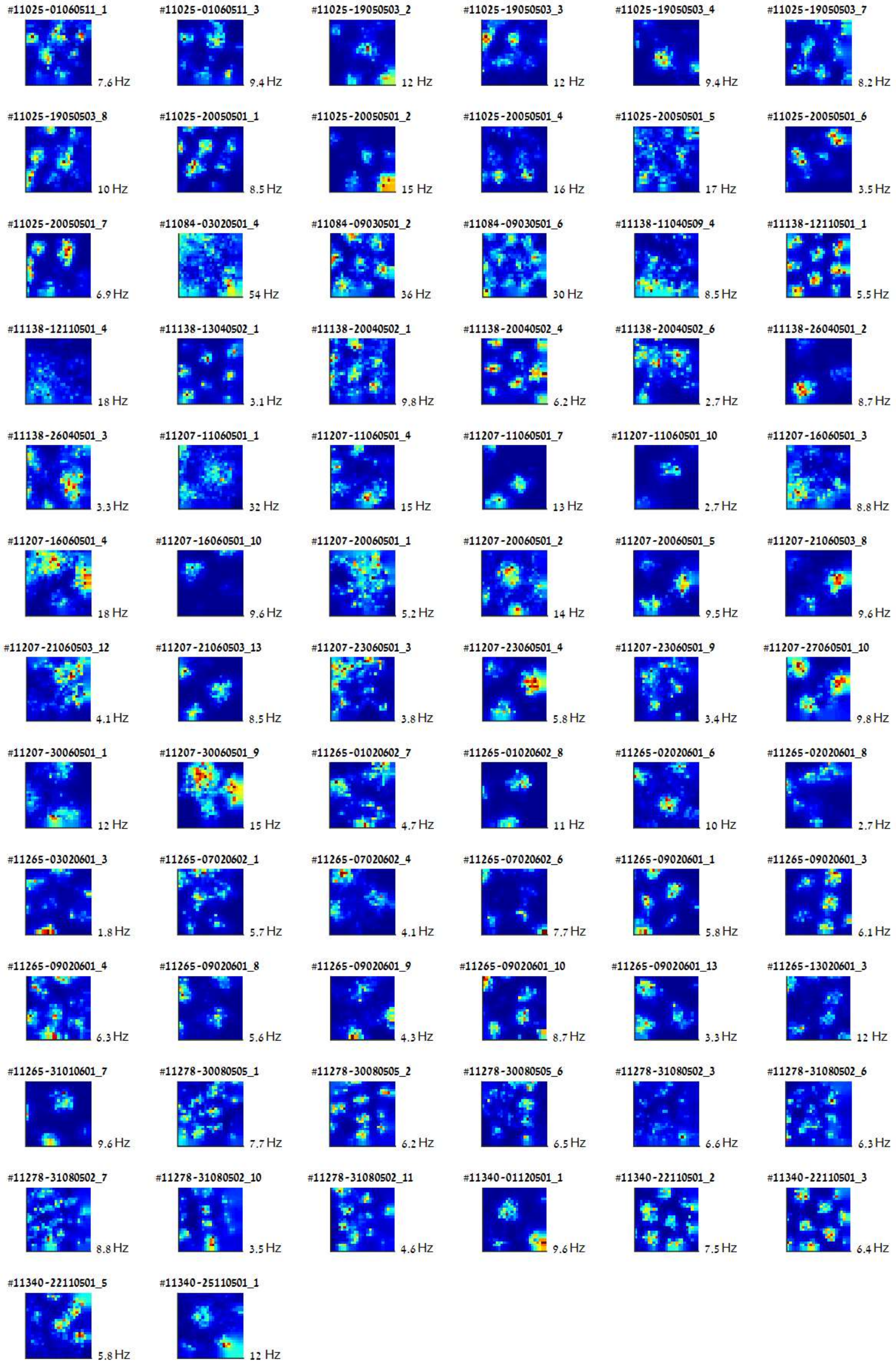

Supplementary Figure 4 – Vinepinsky & Perchik et al

Head direction filters

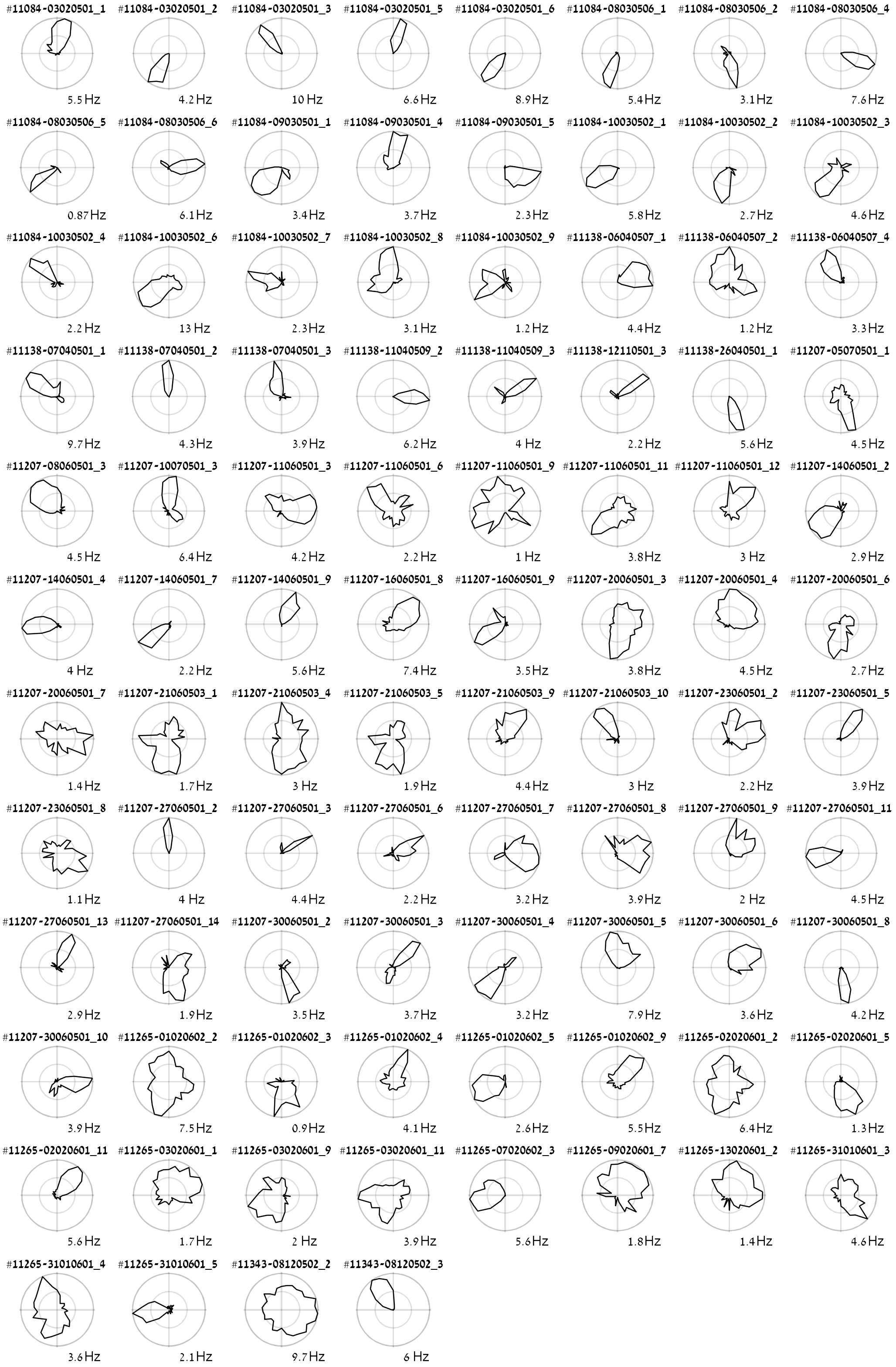

Supplementary Figure 5 – Vinepinsky & Perchik et al

Conjunctive Position and Head direction filters

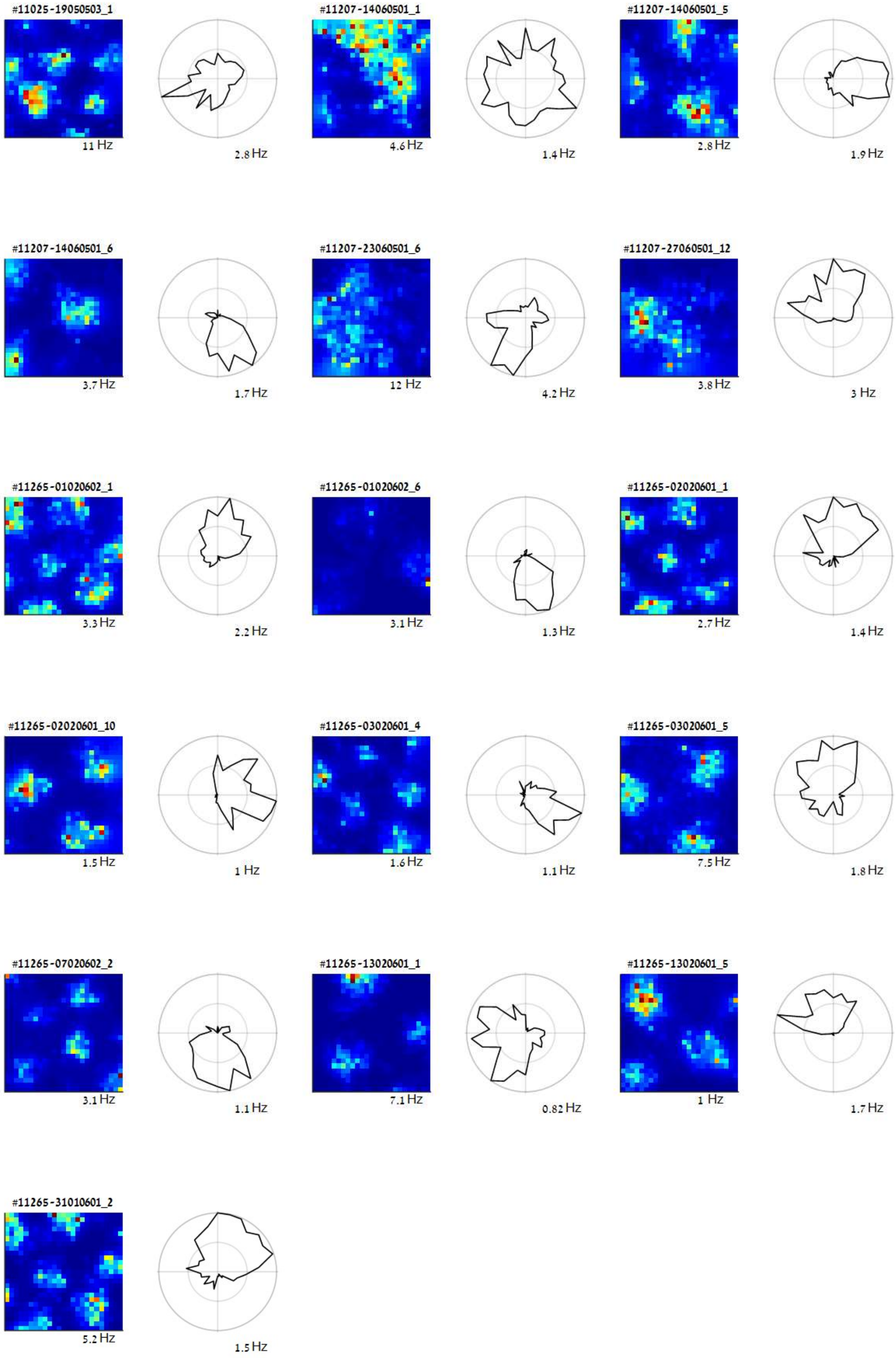

Supplementary Figure 6 – Vinepinsky & Perchik et al

Cell Classification using GLM with Grid, Border and Place Spatial Filters

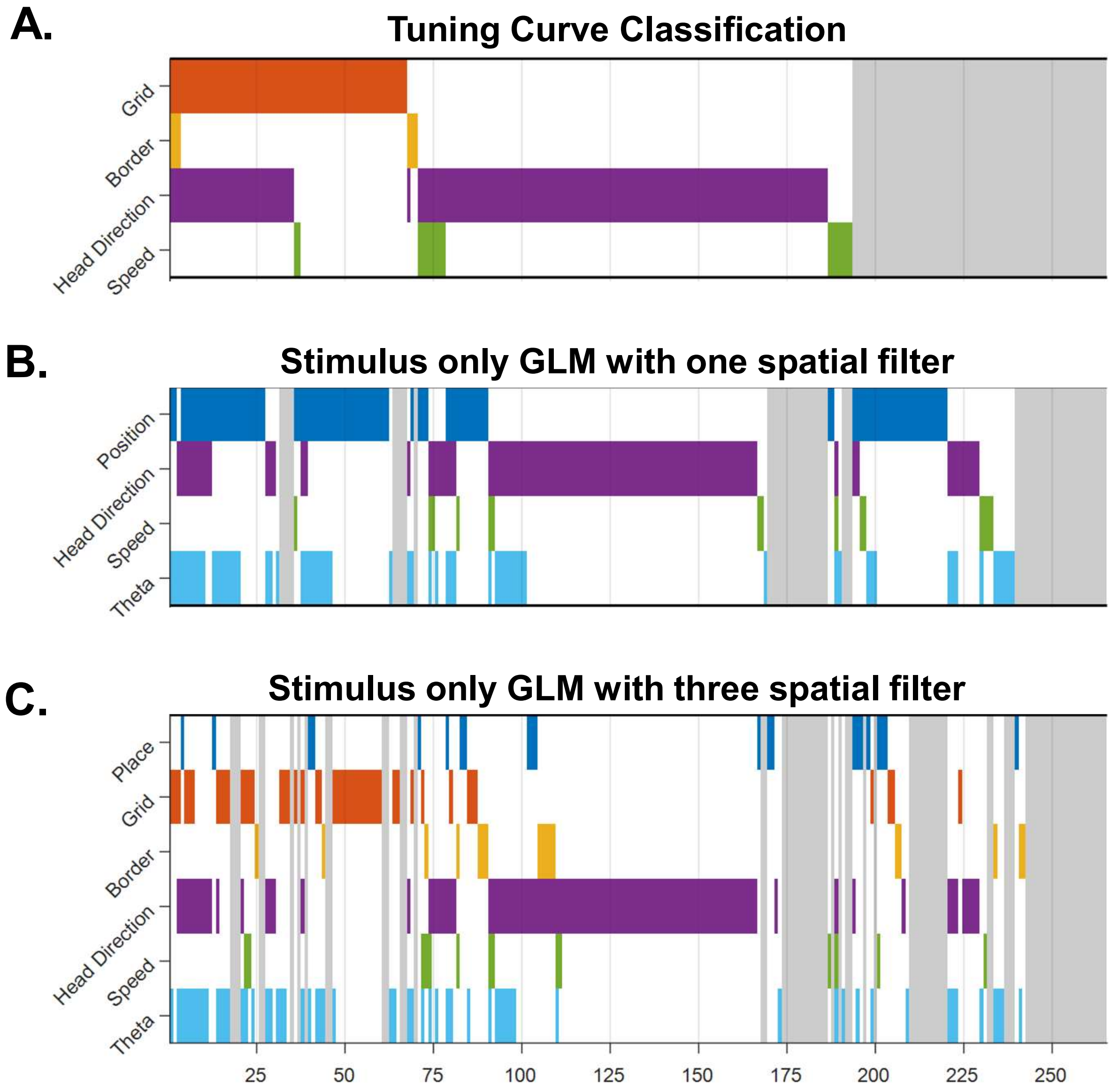

Supplementary Figure 7 – Vinepinsky & Perchik et al
Additional examples of post spike filters

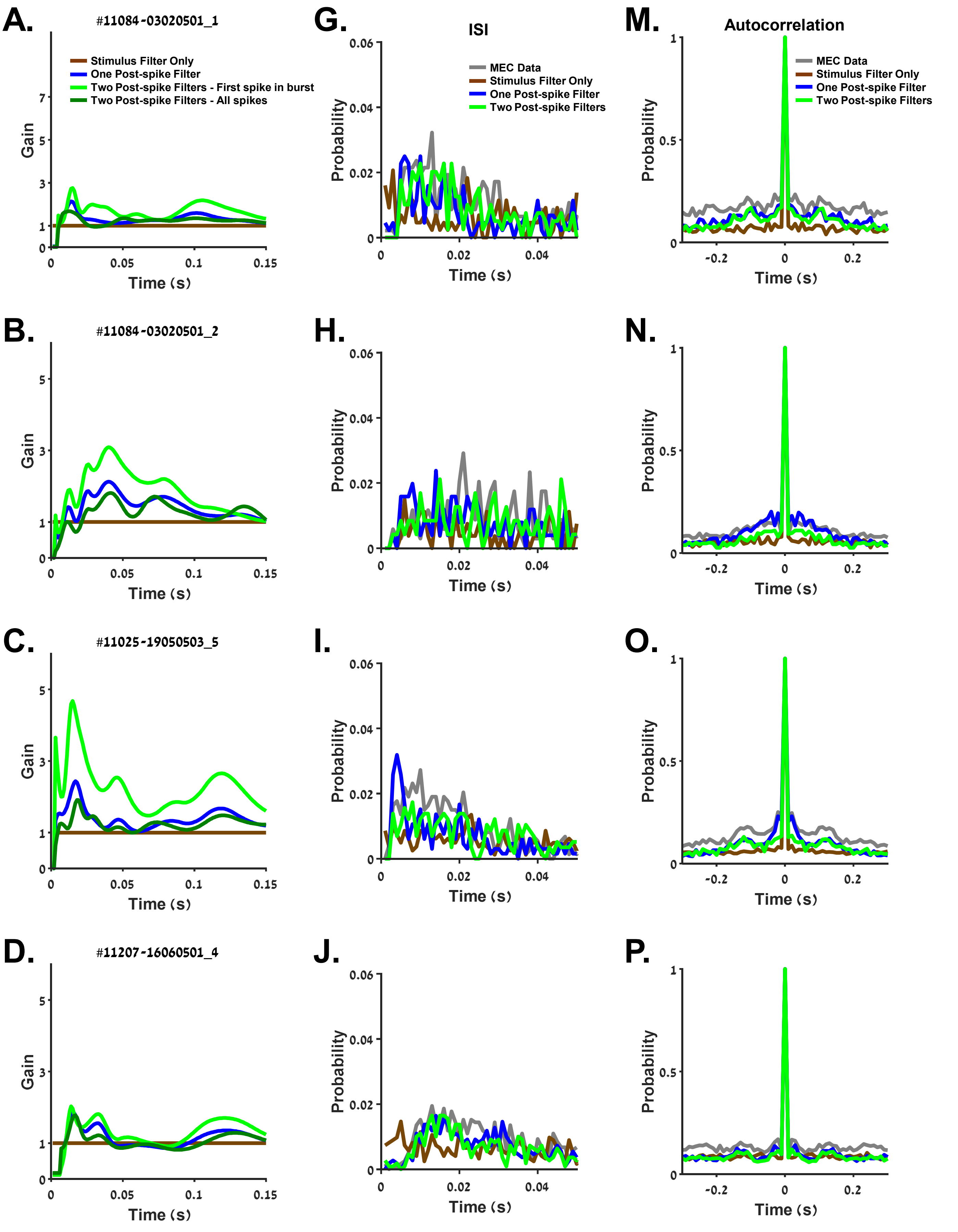

Supplementary Figure 7 – Vinepinsky & Perchik et al
Additional examples of post spike filters

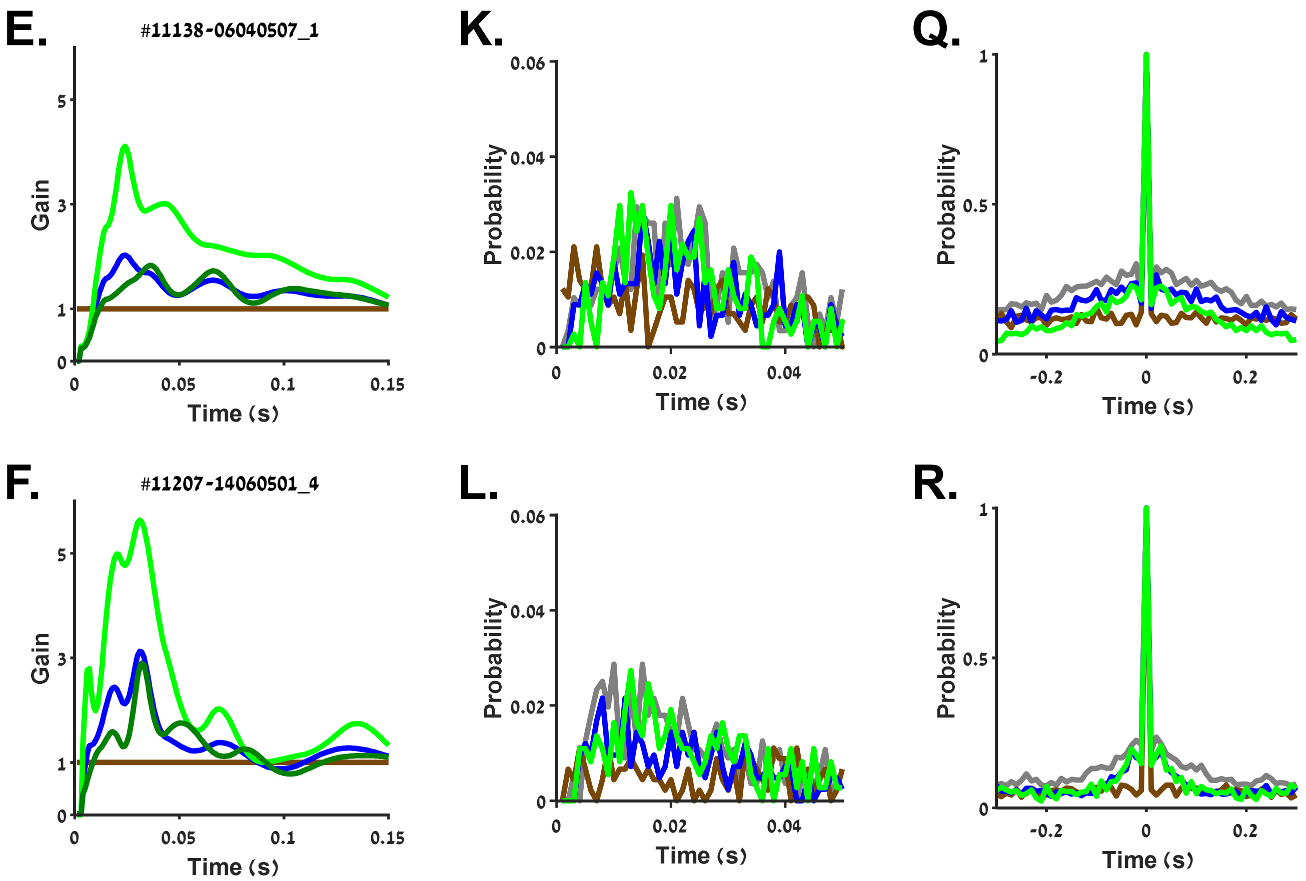

Supplementary Figure 8 – Vinepinsky & Perchik et al
Analysis of two Post-spike filters

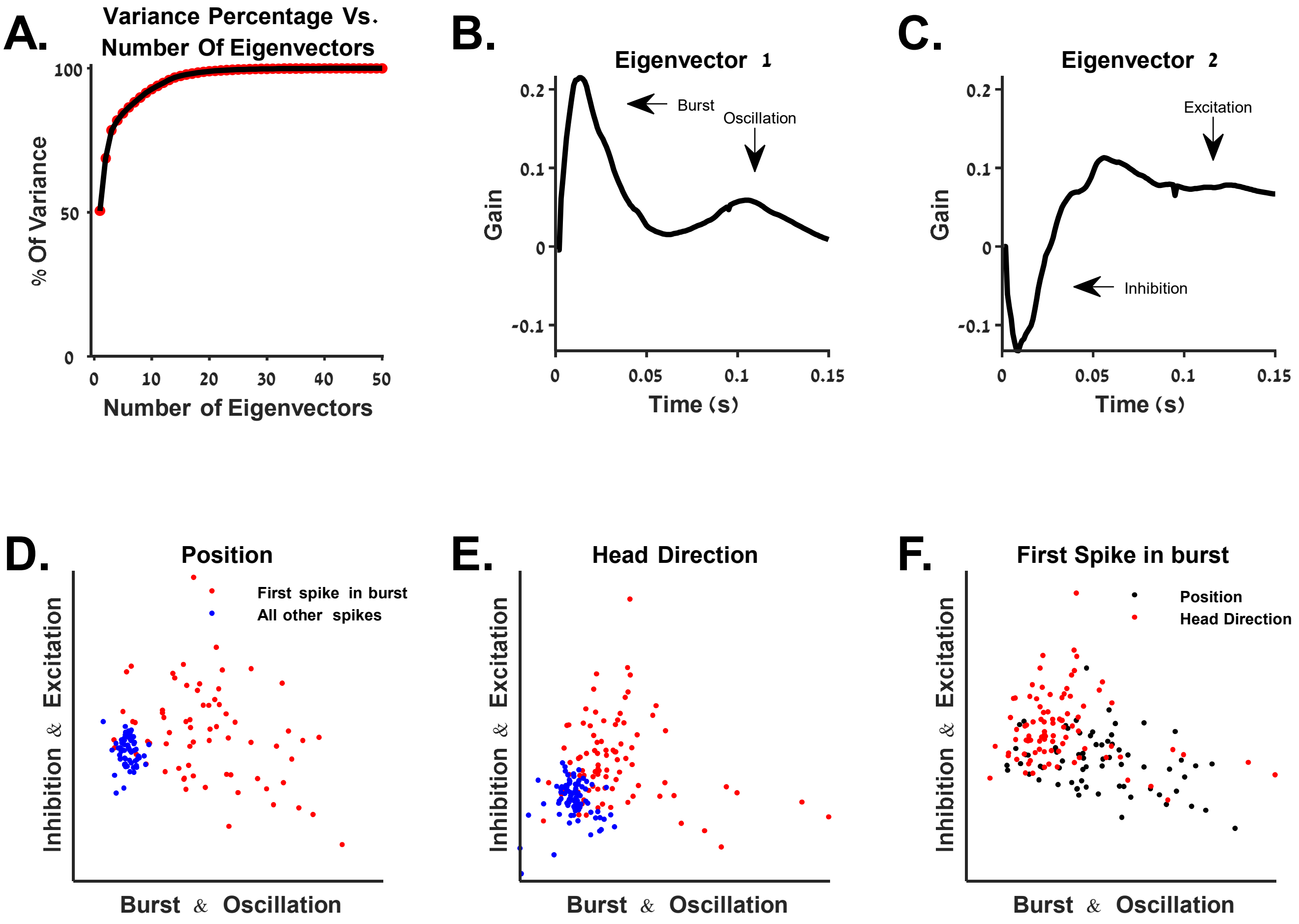

Supplementary Figure 9 – Vinepinsky & Perchik et al
Analysis of population results

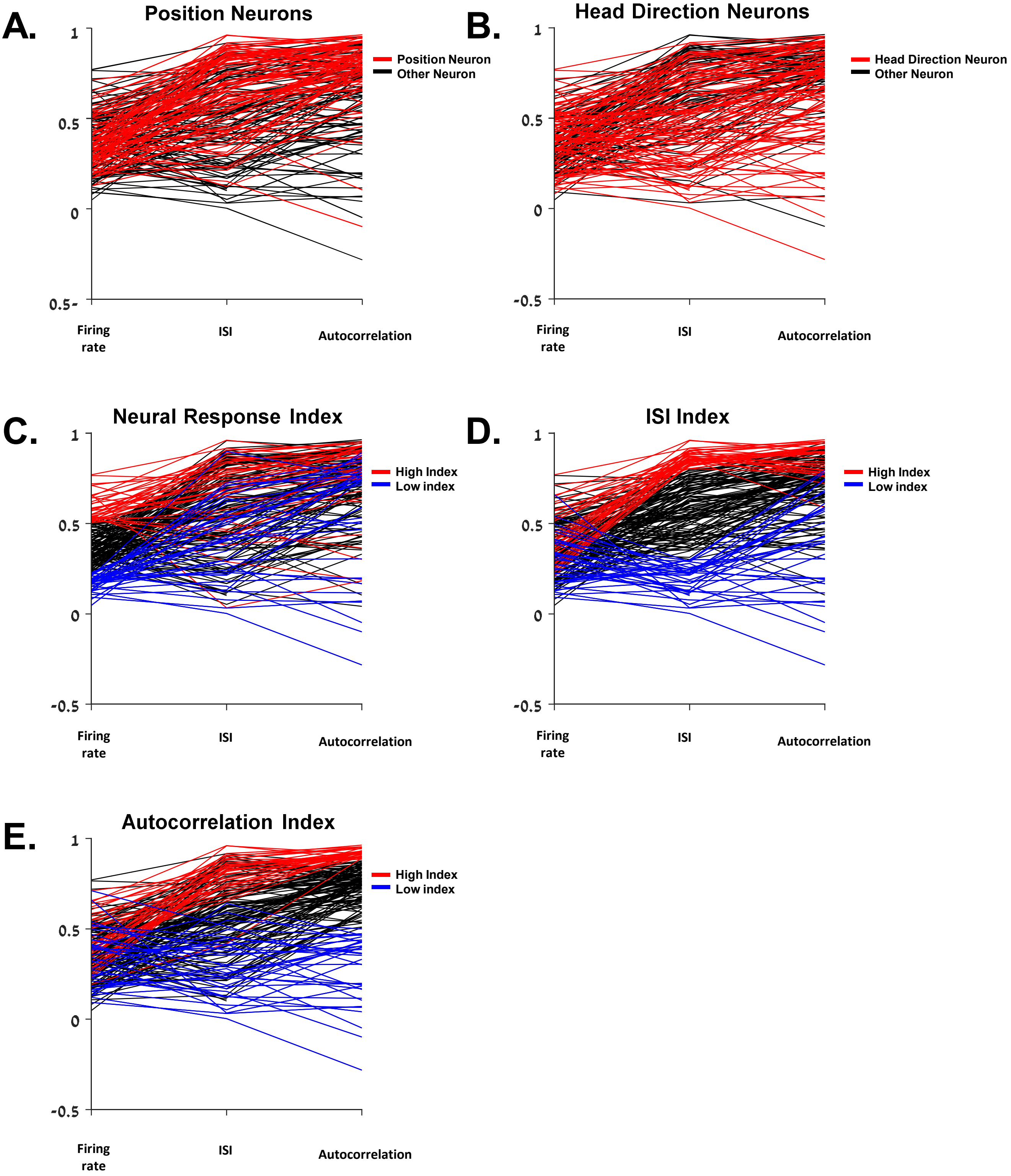

Supplementary Figure 10 – Vinepinsky & Perchik et al
Analysis of simple interaction strength between neurons

A.

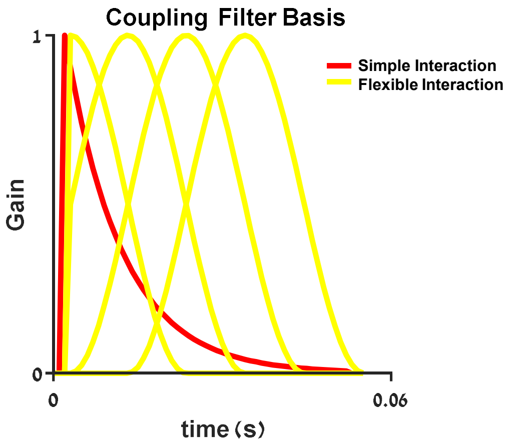

Supplement: Supplementary file 1 [file Data_Sheet_1.pdf]
